# Direct activation of airway sensory C‐fibers by SARS‐CoV‐2 S1 spike protein

**DOI:** 10.14814/phy2.15900

**Published:** 2023-12-20

**Authors:** Joyce S. Kim, Fei Ru, Sonya Meeker, Bradley J. Undem

**Affiliations:** ^1^ Department of Medicine Johns Hopkins University School of Medicine Baltimore Maryland USA

**Keywords:** C‐fiber, cough, sensory nerve, vagus, virus infection

## Abstract

Respiratory viral infection can lead to activation of sensory afferent nerves as indicated by the consequential sore throat, sneezing, coughing, and reflex secretions. In addition to causing troubling symptoms, sensory nerve activation likely accelerates viral spreading. The mechanism how viruses activate sensory nerve terminals during infection is unknown. In this study, we investigate whether coronavirus spike protein activates sensory nerves terminating in the airways. We used isolated vagally‐innervated mouse trachea‐lung preparation for two‐photon microscopy and extracellular electrophysiological recordings. Using two‐photon Ca^2+^ imaging, we evaluated a total number of 786 vagal bronchopulmonary nerves in six experiments. Approximately 49% of the sensory fibers were activated by S1 protein (4 μg/mL intratracheally). Extracellular nerve recording showed the S1 protein evoked action potential discharge in sensory C‐fibers; of 39 airway C‐fibers (one fiber per mouse), 17 were activated. Additionally, Fura‐2 Ca^2+^ imaging was performed on neurons dissociated from vagal sensory ganglia (*n* = 254 from 22 mice). The result showed that 63% of neurons responded to S1 protein. SARS‐CoV‐2 S1 protein can lead to direct activation of sensory C‐fiber nerve terminals in the bronchopulmonary tract. Direct activation of C‐fibers may contribute to coronavirus symptoms, and amplify viral spreading in a population.

## Graphical abstract

We investigated whether the S1 subunit of the β‐CoV spike protein could directly activate sensory C‐fibers. Limited presence of ACE2 or TLR4 in mouse vagal sensory C‐fiber neurons suggests that direct activation doesn't occur via ACE2 or TLR4 binding. Other potential pathways, such as galectin‐3 dependent or TRP channel, warrant further investigation. Regardless of the specific mechanism, these findings suggest coronaviruses may directly trigger respiratory C‐fiber nerve endings, potentially intensifying symptoms and aiding viral spread.

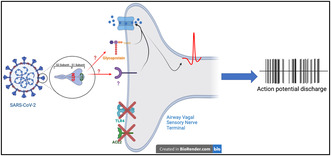

## 1 INTRODUCTION

Most respiratory virus infections lead to the activation of afferent (sensory) nociceptive C‐fibers as evidenced by the nearly ubiquitous sore throat, coughing, sneezing, and increases in reflex parasympathetic secretions that accompany the infections. Each of these symptoms is the expected result of activation of subsets of sensory nociceptive C‐fibers (Mazzone & Undem, 2016). Activation of these nerve endings and the consequent sneezing, coughing, and reflex secretions enable the virus to escape the body and infect another host, i.e., nerve activation amplifies viral spreading. Also, it is well known that respiratory viral infections exacerbate asthma attacks, especially in children; some 80% of asthma‐related emergency room visits by children are associated with a respiratory viral infection (Dulek & Peebles, 2011). Likewise, respiratory viral infections exacerbate COPD (Sethi, 2011). It is likely that the C‐fiber mediated reflex parasympathetic bronchoconstriction and mucus secretions contributes to the exacerbation of these diseases (Zaccone & Undem, 2016).

The β‐coronoviruses (β‐CoVs) are one of four genera (α‐, β‐, γ‐, δ‐) of coronaviruses. Some β‐CoVs are associated with mild “common cold” symptoms and asthma exacerbations. A number of other β‐CoVs have been reported to cause dangerous respiratory infections in humans, including MERS‐CoV, SARS‐CoV, and SARS CoV‐2 (Li, 2016). These coronaviruses express protruding protein “spikes” that decorates the surface of the viral particles. This viral spike (S) protein plays an essential role in binding to the receptors and allowing virus entry into host cells (Huang et al., 2020). S protein is a glycosylated trimer composed of S1 (residues 14–685) and S2 subunit (686–1273 residues). We focused on the S1 subunit in this study, as this subunit is implicated in cell activation. S1 consists of the N‐terminal domain (NTD), C‐terminal domain (CTD), which is also known as the receptor‐binding domain (RBD) (Duan et al., 2020; Seyran et al., 2021). CTD of the S1 subunit (S1‐CTD) binds with high affinity to angiotensin converting enzyme 2 (ACE2) to gain access to cells, and can also activate cells via interactions with toll‐like receptor (TLR4) (Aboudounya & Heads, 2021; Choudhury & Mukherjee, 2020). NTD of the S1 subunit (S1‐NTD) also comprises a so‐called “galectin fold” that has a high degree of homology with galectin‐3 (Gal‐3). This property has been implicated in S1 protein‐induced activation of human monocytes (Schroeder & Bieneman, 2022; Tilkian et al., 1976).

In theory, respiratory viral infections can lead to activation of sensory C‐fibers indirectly or directly. Indirect activation occurs when the virus infects epithelial cells (or other cell types), causing the release of autocoids and mediators that are capable of activating or sensitizing the C‐fiber terminals residing in the airway mucosa. For example, airway C‐fibers have been shown to be activated by viral infection‐associated cytokines and mediators such as type I and type II interferons and sphingosine phosphates (Patil et al., 2019, 2020). Direct activation, by definition, occurs through an interaction between viral particles and C‐fiber terminals. As far as we know, direct activation of airway C‐fibers by viruses has not been rigorously investigated. In this study, three orthogonal approaches were used to study the activation of airway nociceptive C‐fibers by the S1 subunit of β‐CoV spike protein.

## 2 METHODS

### 2.1 Ethical approval

All animal experiments were approved by the Johns Hopkins School of Medicine Animal Care and Use Committee (IACUC; approval reference MO22M69). All guidelines for ethical protocols and care of experimental animals established by the National Institutes of Health (NIH) were followed. We also confirm that we understand the ethical principles under which the journal operates and that our work complies with the animal ethics checklist as outlined in the instructions to authors.

### 2.2 Animals

The animals were housed in an approved animal facility under a 12:12 h light/dark cycle with controlled temperature and humidity, in groups and in cages providing unrestricted access to food and water (ad libitum). We used male mice (C57BL/6J) purchased from Jackson Labs. For two‐photon Ca^2+^ imaging, we used male and female *Pirt‐Cre;R26‐GCaMP6s* mice. The *Pirt‐Cre* mice were generated in X. Dong's laboratory (Han et al., 2018). The ROSA26‐lsl‐GCaMP6s line was originally provided by D. Bergles at The Johns Hopkins University and is not available in Jackson Labs.

### 2.3 Chemicals and reagents

All compounds and drugs used were freshly prepared from stock solutions stored at −20°C. Recombinant human coronavirus SARS‐CoV‐2 spike glycoprotein S1 (Active) was purchased from Abcam (ab273068). α,β‐mATP was purchased from Cayman Chemical (10008956). α,β‐mATP stocks were prepared in millipore water and capsaicin in ethanol. Krebs bicarbonate solution (KBS) was made on the day of the experiment. Dispase II was purchased from Roche (10888700). HBSS (14170120), Leibovitz's L‐15 Medium (11415064), and Fura‐2 am (F1211) were purchased from Thermo Fisher Scientific. Capsaicin (M2028), Collagenase from Clostridium histolyticum (C0130), Laminin from Engelbreth‐Holm‐Swarm murine sarcoma basement membrane (L2020), and Poly‐D‐lysine hydrobromide (P6407) were purchased from Sigma Aldrich.

### 2.4 Extracellular recordings

**FIGURE 1:**
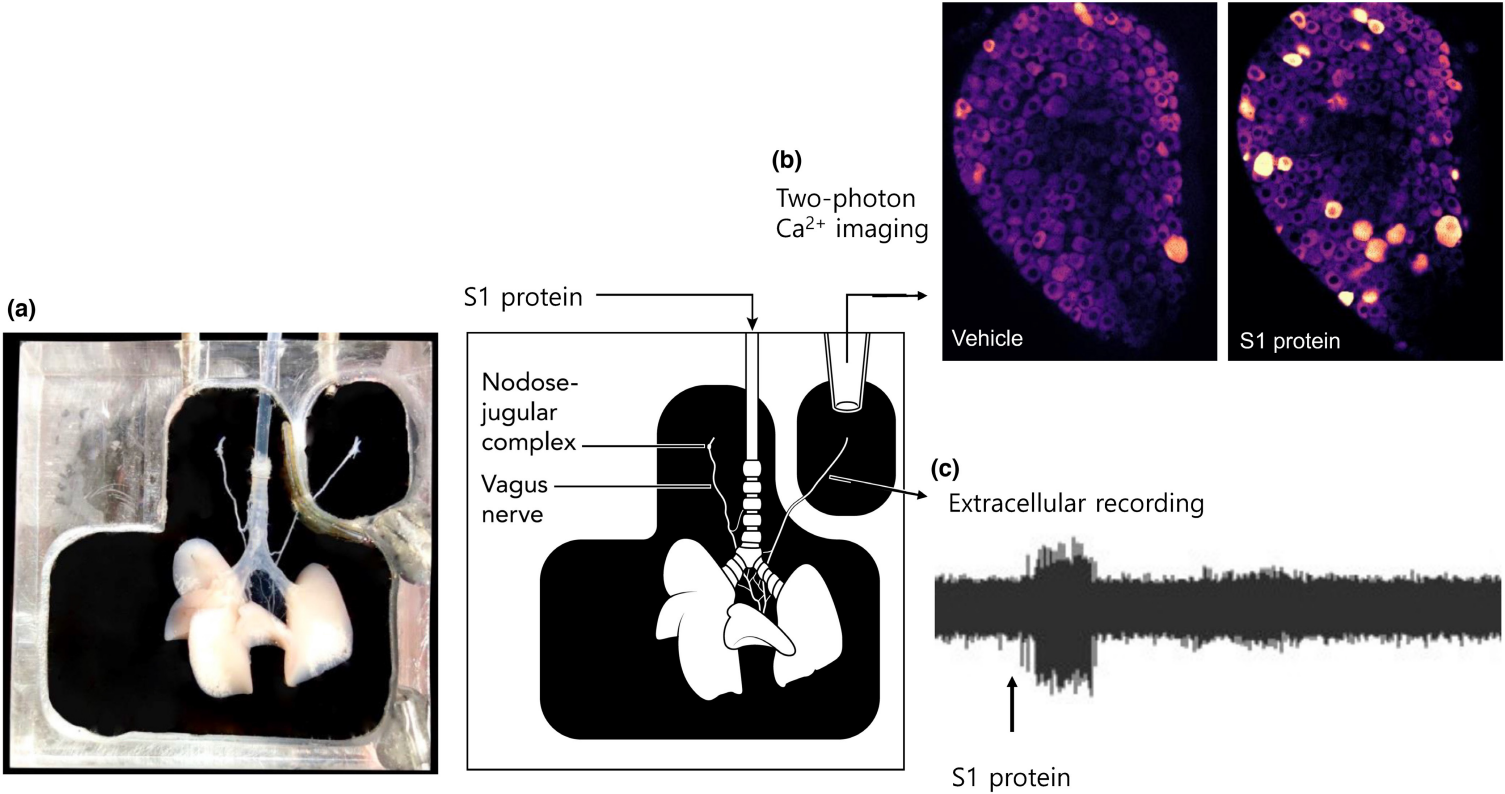
Extracellular electrophysiological recording and two‐photon Ca2+ imaging preparation. (a) Photograph and schematic of an ex‐vivo vagus‐innervated mouse trachea‐lungs preparation for extracellular electrophysiological recording techniques or two‐photon Ca^2+^ imaging. (b) images of one out of the 10 planes of the nodose ganglia from Pirt‐Cre;R26‐GCaMP6 mice, recorded using two‐photon microscope showing [Ca^2+^]_i_ increase (bright orange) in nodose ganglia neurons, in the presence of vehicle (1 mL) and S1 protein (4 μg/mL). (c) Representative tracing showing action potential discharge evoked by S1 protein (4 μg/mL) in nodose C‐fiber terminating in the mouse lung.

The vagus‐innervated trachea‐lung preparation was prepared (Figure 1a), mounted in a two‐chamber bath, and constantly superfused with KBS composed of (mM) 118 NaCl, 5.4 KCl, 1.0 NaH_2_PO_4_, 1.2 MgSO_4_, 1.9 CaCl_2_, 25.0 NaHCO_3_, and 11.1 dextrose (gassed with 95%O_2_–5%CO_2_, pH 7.4, 37°C), as previously described (Kollarik et al., 2003). Action potentials were recorded at the level of the cell body using a sharp extracellular glass electrode (impedance 2 MΩ when filled with 3 M NaCl) strategically positioned in the nodose ganglion. The recorded action potentials were amplified (Microelectrode AC amplifier 1800; A‐M Systems, Everett, WA, USA), filtered (0.3 kHz of low cut‐off and 1 kHz of high cut‐off), and monitored on an oscilloscope (TDS320; Tektronix, Beaverton, OR, USA) and a chart recorder (TA240; Gould, Valley View, OH, USA). The scaled output from the amplifier was captured and analyzed by a Macintosh computer using NerveOfIt software (Phocis, Baltimore, MD, USA). For measuring conduction velocity, electrical stimulation (S44; Grass Instruments, Quincy, MA, USA) was applied to the core of the receptive field. The conduction velocity was calculated by dividing the distance along the nerve pathway by the time delay between the shock artifact and the action potential evoked by electrical stimulation. When a C‐fiber (<1 ms^−1^) was found, the recording was started. For chemical stimulation, 1 mL of vehicle, S1 protein (4 μg), α,β‐mATP (10 μM), or capsaicin (CAP; 0.3 μM) was injected (bolus) into the lung through the trachea.

### 2.5 Two‐photon live imaging

We use the same isolated innervated airway preparation as we do for our electrophysiological studies, except that we employ two‐photon microscopic imaging at the ganglion. For this, Dr. Xingzhong Dong et al in the Department of Neuroscience at Johns Hopkins University, developed *Pirt‐Cre;R26‐GCaMP6s* mice in which the pan‐sensory neuronal *Pirt* promoter drives the expression of GCaMP6s only in sensory neurons (Han et al., 2018). The genetically encoded GCaMP6s is a highly sensitive fluorescent calcium indicator. When action potentials from the peripheral terminals are conducted to the CNS, they also invade the cell soma in the sensory ganglion. When the action potential occurs at the somal membrane it activates voltage‐gated calcium channels that allow calcium to enter the cell. We have noted that sufficient calcium enters the cell with a single action potential to activate GCaMP6s with intensities detectable by our two‐photon microscope. We have carried out extensive electrical stimulation analyses to show that the intensity of fluorescence depends on action potential number in a linear fashion between 1 Hz and 10 Hz stimulation (when given in 5 s trains). To quantify the action potentials, we acquire live images of the ganglia at 10 frames per 6 s at a depth of approximately 100 μm (10 planes of 10 μm thick slices are imaged). It was considered that neurons that projected to the airways are either those with baseline activity or those that respond to given stimuli. The number of “responding neurons” are counted as those that have fluorescence intensity of >1.5‐fold over baseline. The strength of the response is quantified as the average of the peak fluorescence intensity of each responding neuron (Patil et al., 2019, 2020). One milliliter of vehicle, S1 protein (4 μg), α,β‐mATP (10 μM), or capsaicin (CAP; 0.3 μM) was injected (bolus) into the lung through the trachea. In some cases, the slight mechanical perturbation caused by the 1 mL infusion may activate low threshold mechanosensors. Any cell activated by the vehicle control is removed from the study.

### 2.6 Dissociation of mouse nodose neurons

The isolation of nodose neurons has been previously described (Kim et al., 2023). The animals were euthanized by 100% CO_2_ (a fill rate of 50% chamber vol/min) asphyxiation. We observed each mouse for lack of respiration and withdrawal reflex. Once both signs were observed, the nodose ganglia (the bottom distal 2/3 of the ganglia) were dissected and cleared of adhering connective tissue. Isolated ganglia were incubated in an enzyme buffer (2 mg collagenase and 2 mg dispase II in 1 mL Ca^2+^ and Mg^2+^ free Hanks' balanced salt solution) for 60 min at 37°C. Neurons were dissociated by trituration with three fire‐polished glass Pasteur pipettes of decreasing tip pore size at the end of 30, 45, and 60 min. One milliliter enzymatic solution containing the dissociated neurons was suspended in pre‐warmed 10 mL Leibovitz's L‐15 medium containing 10% fetal bovine serum (FBS) and centrifuged at 600*g* for 2 min. After one more wash with L‐15 medium (10% FBS), the cell suspension was transferred onto poly‐D‐lysine/laminin‐coated coverslips and incubated at 37°C for 2 h. After the suspended neurons had adhered to the coverslips for 2 h, the neuron‐attached coverslips were flooded with the L‐15 medium (10% FBS) and used within 8 h for Fura‐2 calcium imaging.

### 2.7 Fura‐2 calcium imaging

Cover slips with adhered dissociated nodose neurons were loaded with Fura‐2 am (5 μM, Invitrogen, Carlsbad, CA) for 45 min at 37°C under an atmosphere of 5% CO_2_‐95% air. Following incubation, we replaced the medium with calcium‐free Hank's Balanced Salt Solution (HBSS) composed of (mM) 130 NaCl, 5 KCl, 2.56 MgCl_2_, 10 HEPES, 16.64 Dextrose with 1.5 mM external Ca^2+^, then mounted in a closed polycarbonate chamber clamped in a heated aluminum platform (PH‐2; Warner Instrument, Hamden, CT) on the stage of a Nikon TSE 100 Ellipse inverted microscope (Nikon, Melville, NY). Chamber temperature was maintained at 37°C with an in‐line heat exchanger and dual‐channel heater controller (models SF‐28 and TC‐344B; Warner Instrument). Fura‐2 am undergoes a change in its fluorescence excitation wavelength, shifting from 380 to 340 nm when it binds to Ca^2+^. The emitted light from the cell was then captured through the objective lens and detected by an imaging camera. An electronic shutter was used to minimize photobleaching of dye. Experimental procedures were carried out, and data acquisition was performed using InCyte software (Intracellular Imaging, Cincinnati, OH). [Ca^2+^]_i_ levels were calculated from an in vitro calibration curve, so they should be regarded as an approximation of the true [Ca^2+^]_i_. The number of “responding neurons” are counted as those that have fluorescence intensity of >1.1‐fold over baseline.

### 2.8 Analysis

Data are expressed as mean ± S.D. The *n*‐value in the extracellular recording studies reflects the numbers of C‐fibers studied with 1 C‐fiber studied per mouse. Student's *t*‐test for paired observations was used to evaluate the statistical significance of the difference between mean values in the control and stimuli.

For multiple comparison, we used ordinary one‐way ANOVA with Turkey's multiple comparisons test, with a single pooled variance.

## 3 RESULTS

### 3.1 Airway afferents are acutely activated by S1 protein

**FIGURE 2:**
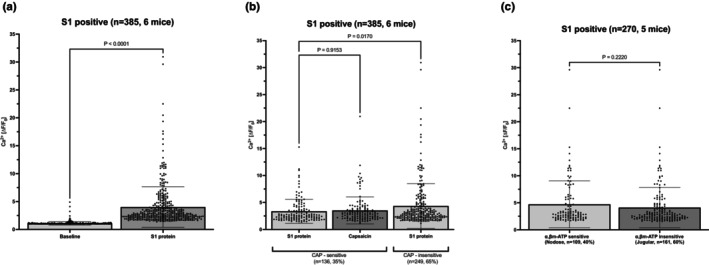
S1 protein activates vagal sensory neurons in two‐photon microscopy. (a). Bar graphs showing mean intensity values of S1 positive neurons at baseline (1.1 ± 0.3, *n* = 385) and response to S1 protein (4.0 ± 3.6, *n* = 385) with a calcium increase >1.5 over baseline. (b) Bar graphs showing mean intensity values of S1 positive neurons in response to Capsaicin (3.5 ± 2.5, *n* = 136) and S1 protein in CAP‐sensitive neurons (3.4 ± 2.2, *n* = 136) and CAP‐insensitive neurons (4.3 ± 4.2, *n* = 249). (c) Bar graphs showing mean intensity values of S1 protein in α,β‐mATP‐sensitive neurons (4.7 ± 4.3, *n* = 109) and α,β‐mATP‐insensitive neurons (4.1 ± 3.7, *n* = 161). Mean ± SD. The *p*‐value was determined by paired two‐tailed *t*‐test and ordinary one‐way ANOVA with Tukey's multiple comparisons test, with a single pooled variance.

We first evaluated whether S1 protein evoke acute activation of vagal sensory nerves using two‐photon Ca^2+^ imaging. Two‐photon microscopy combined with the vagally innervated trachea‐lung preparation (Figure 1a, b) provides for a method to evaluate the activity of more than 100 vagal afferent nerves in a single experiment. We used this technology to evaluate 786 neurons from 6 *Pirt‐Cre;R26‐GCaMP6s* mice. In a previous study it was noted that a concentration of 5 μg/mL of S1 protein led to immune cell activation independently of ACE2 (Schroeder & Bieneman, 2022). We aimed to determine a minimum effective concentration starting at 1 μg/mL. Whereas 1 μg/mL had no effect, 4 μg/mL caused a substantive effect on the sensory C‐fibers. Due to cost prohibitions, larger concentrations were not evaluated. It should be kept in mind, that this concentration of S1 protein in a 1 mL volume is slowly perfused into the mouse lung that is constantly superfused with fresh buffer at a rate of ~4 mL/min. Therefore, the concentration of the S1 protein at the nerve terminals is some unknown fraction of 4 μg/mL. When S1 protein (4 μg/mL) was applied to the receptive field in the airways, 385 neurons (49%) responded with an intensity of >1.5‐fold over baseline (Figure 2a). Vagal sensory neurons innervate the respiratory tract comprise both fast conducting A‐fibers and slowly conducting C‐fibers. With the imaging technique, information on conduction velocity of the responding nerves is not available, but we have previously noted that virtually all mouse bronchopulmonary fibers that respond to capsaicin are slowly conduction C‐fibers (though not all C‐fibers respond to capsaicin) (Kollarik & Undem, 2004). We evaluated the overlap between S1 protein‐sensitive and capsaicin sensitivity and noted that ~35% of the S1 sensitive nerves were subsequently stimulated by capsaicin (Figure 2b). The intensity of the response to the S1 protein, a rough estimate of the number of action potentials arising at nerve cell body, was modestly but significantly larger, in the capsaicin‐insensitive population of nerves (Figure 2b).

Nodose, fibers in the respiratory tract can be distinguished from jugular C‐fibers functionally in that they are selectively activated by the P2X3 and P2X2,3 selective agonist α,β‐mATP. We noted that both α,β‐mATP‐sensitive (presumed nodose fibers) and α,β‐mATP‐insensitive fibers (presumed jugular fibers) were similarly activated by the S1 protein (Figure 2c).

**FIGURE 3:**
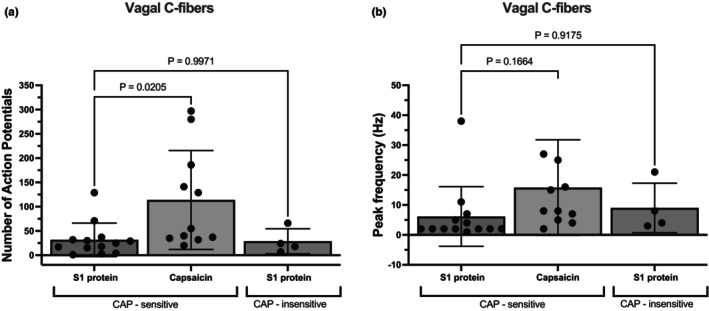
S1 protein acutely evoked action potential discharge in sensory C‐fibers. (a) Bar graphs showing total number of action potential discharge of S1 positive neurons in response to capsaicin (0.3 μM) and S1 protein in CAP‐sensitive (32 ± 35, *n* = 13) and CAP‐insensitive (29 ± 26, *n* = 4) vagal C‐fibers. Capsaicin (0.3 μM) evoked 114 ± 102 action potential (*n* = 11). (b) Bar graphs showing the peak frequency of S1 positive neurons in response to capsaicin (0.3 μM) and S1 protein in CAP‐sensitive (6 ± 10 Hz, *n* = 13) and CAP‐insensitive (9 ± 8 Hz, *n* = 4) vagal C‐fibers. Capsaicin (0.3 μM) caused peak frequency of 16 ± 16 Hz (*n* = 11). Mean ± SD. The *p*‐value was determined by ordinary one‐way ANOVA with Tukey's multiple comparisons test, with a single pooled variance.

Next, we used extracellular electrophysiological nerve recordings to evaluate S1 protein‐induced activation of bronchopulmonary sensory nerves (Figure 1c). In general, only one nerve was studied per mouse (*n* = number of fiber per animal). Although the experiments are demanding, the electrophysiological recordings are ideally suited for providing detailed information on the conduction velocity (CV) of the fiber activated as well as the number, frequency, and pattern of action potential discharge in response to a stimulus. We evaluated 39 pulmonary C‐fibers (CV = 0.6 ± 0.1). In these experiments, all but four of the fibers studied were presumed nodose C‐fibers. When S1 protein (4 μg/mL) was applied to the receptive field in the airways, 44% of the C‐fibers (*n* = 17) responded acutely with action potential discharge (Figure 3). As with the imaging studies, we noted that both capsaicin‐sensitive and ‐insensitive terminals were activated by the S1 protein. We quantified the data as the total number of action potentials evoked and the peak frequency (Hz), determined by the maximum number of action potentials in each 1 s bin. Perfusion of trachea with 1 mL of S1 protein (4 μg) evoked 32 ± 35 action potential in capsaicin‐sensitive C‐fibers (*n* = 13) and 29 ± 26 in capsaicin‐insensitive C‐fibers neuron (*n* = 4), *p* = 0.997. The peak frequency of action potential discharge in these C‐fibers averaged 6 ± 10 Hz and 9 ± 8 Hz, respectively; *p* = 0.918. This finding suggests that the overall number of action potentials and the peak rate at which the individual action potentials discharge remains comparable in both groups. For a comparison, capsaicin at its maximally effective concentration, evoked more action potentials (114 ± 102) than the S1 protein, but the peak discharge frequency was similar between the two stimuli (Figure 3b).

### 3.2 S1 proteins can directly activate vagal neurons

**FIGURE 4:**
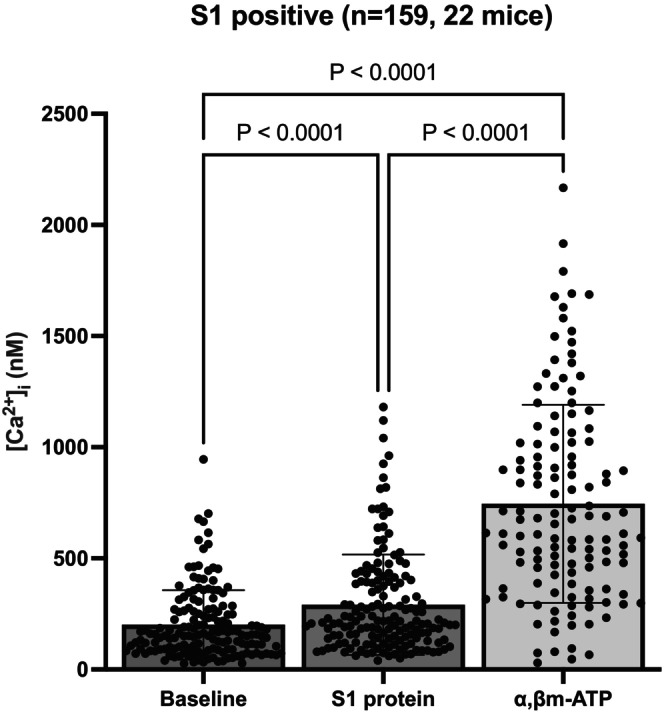
S1 protein can directly activate airway C‐fiber neurons independently of airway tissues. Bar graph showing values for resting [Ca^2+^]_i_ in dissociated mouse nodose neurons (*n* = 159, 201 ± 155) and values of [Ca^2+^]_i_ in response to S1 protein (*n* = 159, 291 ± 226), and α,β‐mATP (745 ± 446,10 μM). Out of 254 neurons in 22 mice, 63% of the isolated neurons (*n* = 159) responded to S1 protein and out of 196 neurons in 20 mice, 65% of the isolated neurons (*n* = 128) responded to α,β‐mATP with a calcium increase >1.1 over baseline. Mean ± SD. The *p*‐value was determined by ordinary one‐way ANOVA with Tukey's multiple comparisons test, with a single pooled variance.

To determine if the stimulation by spike protein was indirect, i.e., requiring non‐neuronal cells the airway tissue, we evaluated its effect on neuronal cell bodies dissociated from the vagal ganglia. The readout in this experiment was a rapid elevation in intracellular calcium using the Fura‐2 as the calcium indicator. We evaluated a total number of 254 neurons in 22 mice. About 63% of the isolated neurons (*n* = 159) responded to spike protein with a calcium increase of >1.1‐fold over baseline (Figure 4). These data support the hypothesis that the spike protein can directly activate neurons independently of airway epithelial cells.

## 4 DISCUSSION

The vagal afferent nerves innervating the respiratory tract comprise those derived from nodose ganglion neurons and the jugular ganglion. The nodose and jugular nerves have different embryological origins and distinct heterogeneity in phenotypes, neurochemistry, and anatomical projections to the respiratory tract and brain stem (Mazzone & Undem, 2016). Different types of C‐fibers exist within the airway nervous system, and the specific subtype of C‐fibers activated can have a significant impact on the outcomes observed, including coughing, dyspnea, and parasympathetic reflexes. For a better understanding of the mechanisms behind these physiological responses, it is crucial to identify which C‐fiber subtypes are activated by the virus. We therefore evaluated whether the terminals of nodose neurons, jugular neurons or both were activated by the S1 protein.

In the mouse, the nodose ganglion (placode derived neurons) and jugular or supranodose ganglion (neural crest‐derived neurons) are often fused into a vagal ganglion complex with the rostral pole of the complex more jugular than nodose and the caudal pole more nodose than jugular (Nassenstein et al., 2010). Therefore the % of nodose and jugular neurons sampled will arbitrarily depend on the positioning of the lens of the microscope. In the mouse and guinea pigs, nodose C‐fibers can be distinguished from jugular C‐fibers functionally in that they are selectively activated by ATP, or the P2X3, and P2X2,3 selective agonist α,β‐mATP (Kwong et al., 2008; Nassenstein et al., 2010; Undem et al., 2004). When we further evaluated the effect of α,β‐mATP, the two‐photon Ca^2+^ imaging showed that the S1 protein led to the stimulation of nodose fibers (α,β‐mATP‐sensitive fibers), as well as those presumed to be jugular fibers.

The rapid onset of activation of the afferent terminals, along with the finding that the S1 protein could rapidly activate sensory nerve cell bodies isolated and from the sensory ganglia and suspended in buffer solution in a manner that diminishes secondary effects of non‐neural cells, support the hypothesis that the nerve activation was a direct effect. These data also prove that airway epithelial cells or other airway cells are not required for the spike protein‐induced sensory nerve activation.

The data provide evidence that SARS‐CoV‐2 spike protein is capable of activating bronchopulmonary C‐fibers with intensities that approximate that seen with capsaicin. The mechanism(s) of how SARS‐CoV‐2 directly activates nerve terminals remain unknown. S1‐CTD binds to ACE2 as well as TLR4 (Aboudounya & Heads, 2021; Choudhury & Mukherjee, 2020). However, RNAseq data reveals little evidence of ACE2 or TLR4 expression in mouse vagal sensory C‐fiber neurons (Wang et al., 2017), including those that innervate the airways (Mazzone et al., 2020). Moreover, the extent to which the S1 protein binds to mouse ACE2 is questionable (Wan et al., 2020). TRP channels in bronchopulmonary C‐fibers, in particular TRPV1 and TRPA1, can be stimulated by disparate stimuli to evoke action potential discharge (Mazzone & Undem, 2016; Veldhuis et al., 2015). The observation that the S1 protein was effective in capsaicin‐sensitive and capsaicin‐insensitive nerves argues that TRPV1 is not essential for activation. In the mouse, virtually all capsaicin‐insensitive fibers are also insensitive to TRPA1 activation (Nassenstein et al., 2008). Therefore, the data also argue against an essential role of TRPA1 is S1‐induced activation.

Another mechanism for S1 protein‐induced cell activation involves Gal‐3. The S1‐NTD contains what is often called the “galectin fold”, due to its structural similarity to human Gal‐3 (Behloul et al., 2020). Schroeder et al., have proposed a Gal‐3 dependent mechanism for S1 protein‐induced cellular (monocyte) activation (Schroeder & Bieneman, 2022). Although galectin signaling can lead to down‐stream ionic modulation in cells (She et al., 2020), the hypothesis that Gal‐3 is involved in C‐fiber stimulation remains speculative until detailed biophysical studies are carried out at the level of patch‐clamp neuronal recordings.

Physiologically, coughing contributes to the body's defense system, but it can also act as a mechanism for infection transmission and may contribute to the pathology of the disease. Additionally, asthmatic children with acute asthma exacerbations are almost entirely caused by respiratory viruses. Viral infection is associated with COPD exacerbations, and their outcome is severe, including decreased lung function, coughing, and prolonged hospitalizations (Dulek & Peebles, 2011). Irrespective of the mechanism, the findings reveal a possibility that coronaviruses may directly stimulate respiratory C‐fiber nerve terminals, an effect that would contribute to symptoms and likely amplify viral spreading. The findings that the spike protein can activate sensory C‐fibers may also contribute to local symptoms of vaccinations with modified S1 protein mRNA. However, further studies are needed to determine whether S1 protein associated with respiratory viral infection or vaccination reach concentrations in the nerve terminal “biophase” to cause action potential discharge.

## FUNDING INFORMATION

This work is supported by NIH NHLBI R35 HL155671 (BJU) and NHLBI F32 HL170490 (JSK).
